# Venous thromboembolism (VTE) risk assessment completion in psychiatric inpatients

**DOI:** 10.1192/bjo.2021.296

**Published:** 2021-06-18

**Authors:** Harriet Powell, Josie Jenkinson

**Affiliations:** 1 Ashford and St Peter's NHS Trust; 2 Surrey and Borders Partnership NHS Foundation trust

## Abstract

**Aims:**

To audit VTE risk assessment compliance across psychiatric inpatient wards at three different sites within Surrey and Borders Partnership NHS Foundation Trust (SABP), and to highlight the importance of completing VTE risk assessments for psychiatric inpatient safety and care as set out by NICE guidelines (2019).

**Method:**

Numbers of VTE risk assessments completed (within 24 hours, and those completed any time during inpatient stay) and VTE risk assessments not completed were collected via SABP electronic mental health records. Percentage compliance for each ward and hospital involved in the study were calculated. Chi square statistical t tests were conducted using Excel to check for associations between type of ward (older adult and working age) and VTE risk assessment completion.

A total of 3004 patients were included in the study. Ages ranged from 18–82 years of age, and both males and females included in the study. A total of 2060 were working age (WA) patients (aged 18–64 years) and 944 were older adults (OA) (aged > 65 years).

**Result:**

Across all three sites, more than 90% of all inpatients admitted between May 2018 and October 2020 did not have a formal VTE risk assessment completed. Across all sites, less than 1% of all inpatients had a completed VTE risk assessment done within 24 hours, as recommended by the NICE guidelines. Older Adult wards showed better compliance with VTE risk assessment completion with 38% of patients on one OA ward having had a completed VTE risk assessment, and 28% on another completed OA ward. Being admitted to an OA ward was strongly associated with VTE risk assessment completion (p < 0.05).

**Conclusion:**

OA wards have hosted QI programmes with regards to VTE risk assessment which may be why VTE risk assessment was more likely to have been completed on OA wards. VTE risk assessment compliance overall is inadequate across all sites included in the study. Recommendations include further education for all ward staff on how, why and when VTE risk assessment should be completed, greater accessibility of an improved VTE risk assessment form and for QI initiatives on OA wards to be rolled out on WA wards. These findings have been presented and discussed at regional Trust teaching days, and this audit will be repeated in one year.

